# Reinnervated muscle fiber type-grouping-inevitable?

**DOI:** 10.18632/oncotarget.15757

**Published:** 2017-02-27

**Authors:** Tessa Gordon

**Affiliations:** Department of Surgery, The Hospital for Sick Children, Toronto, Ontario, Canada

**Keywords:** muscle fiber-type clumping, motor unit action potential, peripheral nerve injury, partial nerve injury, sprouting, Neuroscience

Muscle fiber-type grouping (‘clumping’) in cross-sections of patient muscle biopsies and the electrophysiological recordings of enlarged motor unit action potentials (MUAPs) are almost universally interpreted as peripheral nerve damage with subsequent reinnervation of denervated muscle fibers [1, 2]. Other electromyographic potentials, including nascent potentials, are indicators of early muscle reinnervation. Yet, after experimental reinnervation of large hindlimb cat muscles, muscle fiber-types and the muscle fibers innervated by a single motor nerve fiber, the muscle unit (MU) fibers, exhibit the mosaic distribution that is typical of all normally innervated muscles. Muscle fiber-type and MU fiber clumping become evident in large muscles only when few motor nerve fibers (20% or less) reinnervate these muscles after nerve transection and surgical repair [6]. These experimental findings in large hindlimb muscles may anticipate that muscle fiber-type and MU sprouting are *not* predictors of nerve damage and muscle reinnervation, as has been assumed.

We addressed this possibility by examining the distributions of fiber-types and MU fibers in small muscles of the rat hindlimb for comparison with those in large muscles. Six months after nerve transection and repair, all the nerve fibers reinnervated the denervated rat muscle but, in contrast to the restored mosaic distribution of reinnervated MU fibers and fiber-types in well reinnervated large cat muscles, the reinnervated rat MU muscle fibers were mostly confined within 1-3 muscle fiber clumps; the MU fibers were clumped in parallel with a corresponding fiber-type grouping in the muscle cross-sections [4].

**Figure 1:**
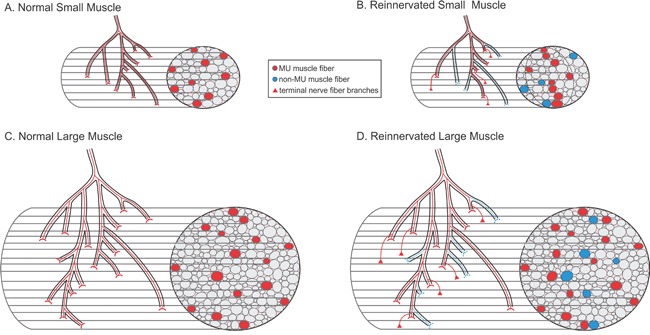
Schematic representation of the intramuscular branching of a single motor nerve fiber and the resultant muscle unit (MU) fiber distribution amongst non-MU fibers.

Each MU muscle fiber is normally surrounded by an average of 6 non-MU muscle fibers and all the MU muscle fibers are confined to a ‘muscle territory’ that is bounded by the outermost MU fibers (Figure 1A, 1C). The territories occupy ~13% of the total muscle cross-sectional area in *both* small and large muscles [4]. The size of the MU territories decreases significantly after reinnervation of small but not the large muscles, the latter having many more muscle fibers. Normally, each motor nerve branches within intramuscular nerve sheathes to distribute the MU muscle fibers amongst non-MU fibers in a mosaic pattern (Figure 1A, 1C). The distal branching is more extensive in the large than the small muscles and the MU fibers are distributed in more muscle fascicles in the large as compared to the small muscles. Our interpretation is that regenerating nerves ‘miss’ some of the most proximal branch points in the intramuscular sheathes (Figure 1B, 1D) with the result that, in the small muscles, the regenerating nerves branch close to the muscle fibers within fascicles to result in MU fiber grouping (Figure 1C). In larger muscles, the longer intramuscular sheathes allow for more widespread distribution of the regenerating nerve fibers through the sheathes and, in turn, there is less requirement for distal nerve fiber branching close to the denervated muscle fibers (Figure 1D).

It is only when few nerve fibers regenerate into a denervated large muscle and each nerve fiber branches to reinnervate 5-8 times as many muscle fibers as normal, that each motor nerve fiber innervates adjacent muscle fibers; MU fibers and fiber-types exhibit clumping within MU territories which do *not* increase in size from normal [6]. This pattern of progressive muscle fiber type and MU muscle fiber clumping in the large reinnervated muscles is the same as the pattern that was observed and described in both large and small muscles after sprouting in partially denervated muscles [5, 7]. The clumping emerges as the regenerating nerves branch close to the denervated muscle fibers and the intact nerve fibers in partially denervated muscles emit sprouts to reinnervate adjacent muscle fibers.

These findings and arguments indicate that fiber-type grouping in reinnervated muscles is not inevitable as has been generally supposed. Fiber-type grouping only occurs in large muscles after extensive partial denervation and when fewer than 20% of the original nerve supply reinnervate muscle after complete nerve transection and surgical repair [6]. In small muscles, fiber-type clumping *is also* indicative of extensive partial denervation as in large muscles [5] but, clumping occurs in reinnervated small muscles after nerve transection and surgical repair even when all or most of the regenerating nerve fibers reinnervate the denervated muscle [4]. Hence fiber-type grouping in small muscles is indicative of *both* sprouting after extensive nerve damage *and* muscle reinnervation after damage that severs all the nerve supply whether or not all the nerves regenerate and reinnervate the denervated muscle. In large muscles on the other hand, fiber-type grouping indicates sprouting after extensive nerve damage whether or not there is partial or complete nerve injury.

The enlarged MUAPs that are frequently recorded from muscles have been and continue to be used as indicators of muscle reinnervation [1, 3]. It is important to note though, that enlarged MUAPs are not, like muscle fiber-type grouping, an invariant result of nerve damage. Rather, these enlarged MUAPs are more likely to correspond with reduced numbers of nerve fibers supplying large muscles. In small muscles, they cannot distinguish between reinnervation after complete or extensive partial nerve injuries, even when reinnervation is excellent after the former injury.
